# Reevaluating the Role and Timing of Fever in Acute Cholecystitis

**DOI:** 10.5811/westjem.63649

**Published:** 2026-09-24

**Authors:** Andrea Villarroel Barrios, Pablo Aguilera Fuenzalida, J. Austin Lee, Valentín Gaete Díaz, Óscar Navea Carrasco, Bárbara Lara Hernández

**Affiliations:** * Pontificia Universidad Católica de Chile, School of Medicine, Department of Emergency Medicine, Santiago, Chile; † Indiana University School of Medicine, Department of Emergency Medicine, Indianapolis, Indiana, USA; ‡ Red de Salud UC CHRISTUS, Department of Emergency Medicine, Santiago, Chile

## Abstract

**Introduction:**

Acute cholecystitis is diagnosed based on clinical and diagnostic findings. Fever is commonly considered a sign of systemic inflammation in acute cholecystitis, although its diagnostic performance may vary depending on the timing of presentation. This study analyzed the prevalence of fever as a diagnostic parameter for acute cholecystitis, according to the symptom onset at the time of medical consultation (≤ 24 hours versus > 24 hours), and its potential association with both medical and surgical complications.

**Methods:**

We conducted a retrospective cohort study in two urban, tertiary care emergency departments (ED) between March 2023 and June 2024. Adult patients (≥ 18 years) with clinically diagnosed acute cholecystitis were included. Presentations were categorized by time since symptom onset: early (≤ 24 hours) or late (> 24 hours). Fever was defined as axillary temperature > 37.5 °C at home or at ED arrival. The primary outcome was the sensitivity of fever for the diagnosis of acute cholecystitis, stratified by early versus late presentation. Secondary outcomes included the sensitivity of other inflammatory markers and the association between fever and medical or surgical complications. The association between fever and complications was evaluated using a penalized logistic regression model (Firth method) to account for the low number of outcome events.

**Results:**

A total of 207 cases of acute cholecystitis were included in the analysis: 108 early presenters; and 99 late presenters. Fever was present in 4.6% (95% CI, 1.5–10.4) of early presenters and 20.2% (95% CI, 12.8–29.4) in late presenters, with an overall sensitivity of 12.1% (95% CI, 8.0–17.2). Medical and/or surgical complications were low in this cohort (n = 15, 7.2%), but more frequent in patients with fever, regardless of time onset of symptom (28% versus 4.5%, *P* < .001), yielding an adjusted odds ratio of 6.64 (95% CI, 1.42–31.21*; P* = .02) after adjusting for leukocyte count, C-reactive protein levels, and age.

**Conclusion:**

Fever is an uncommon sign of acute cholecystitis, especially in early presentations, but may indicate a higher risk for complications when present. These findings underscore the need to reconsider the role of fever in current diagnostic criteria and suggest further validation of its use as a predictor of complications in prospective studies.

## INTRODUCTION

Acute cholecystitis is an acute inflammatory condition of the gallbladder, most often caused by obstruction of the cystic duct by gallstones or biliary sludge (90–95% of cases).^1^ In the United States, 10–15% of adults have gallstones, while the estimated prevalence in the Chilean population is 13.3% in men, and 36.7% in women.^2^,^3^ Acute cholecystitis cases account for up to 10% of all patients with abdominal pain.^4^

The progression from cholelithiasis to acute cholecystitis and its complications depends on the degree and duration of ductal obstruction. This progression is classically described in three stages.^2^ In the initial stage (days 2 to 4), obstruction of the cystic duct causes increased intraluminal pressure and impairs lymphatic drainage; this, combined with bile supersaturation, triggers inflammation, which is reflected as edema and congestion of the gallbladder wall, often observed as wall thickening on imaging. The second stage (days 3 to 5) involves worsening inflammation, which can progress to necrosis, hemorrhage, and potential perforation that may lead to peritonitis. The third stage (day 6 onward) is marked by increase of necrotic tissue, leukocyte infiltration, and infection. Only about 10–30% of acute cholecystitis cases develop secondary infection, underscoring the primary role of obstruction rather than infection in early acute cholecystitis.^5^,^6^

The diagnosis of acute cholecystitis combines clinical, laboratory, and imaging findings, formalized in the Tokyo Guidelines, updated in 2013 and again revised in 2018.^7^ These guidelines define a “suspected diagnosis” of acute cholecystitis as the presence of a local sign of inflammation (Murphy sign, or pain, tenderness, and/or mass in the right upper quadrant) plus a systemic sign of inflammation (fever, elevated C-reactive protein, and/or leukocytosis). A “definitive diagnosis” requires compatible imaging findings, where common imaging modalities include ultrasound, computed tomography (CT), magnetic resonance (MRI) and magnetic resonance cholangiopancreatography (MRCP). Leukocytosis > 18,000/μL is also a marker of moderate severity by these guidelines. Although the derivation study conducted in Japan reported high diagnostic accuracy (sensitivity 92.1%, specificity 93.3%), subsequent studies have shown reduced diagnostic performance in clinical settings, with sensitivities between 83–85% and specificities between 37–50%.^8^,^9^

### Importance

Traditionally, the clinical suspicion of acute cholecystitis relies on the presence of local clinical signs (right upper quadrant pain, nausea, vomiting), along with the presence of fever. This framework has shaped expectations of systemic inflammation as a diagnostic marker, which may reduce the likelihood of early detection of this condition, whose management is essentially surgical, thereby increasing patient morbidity and mortality. Given that acute cholecystitis is primarily an obstructive rather than infectious condition in its initial stages, it may be possible that the lower rates of systemic inflammation observed today result from patients in many settings seeking care earlier than when the Tokyo criteria were originally derived. Exploring this possibility is timely, as it raises questions about the role of systemic inflammation markers in acute cholecystitis diagnosis and severity grading and whether patients presenting earlier in the disease course have different inflammatory profiles than those presenting later.

**Population Health Research Capsule**

What do we already know about this issue?

*Fever is part of the Tokyo Guidelines criteria for acute cholecystitis, but recent studies suggest its diagnostic value may be limited*.

What was the research question?

*Does fever sensitivity for diagnosing acute cholecystitis vary with time from symptom onset?*

What was the major finding of the study?

*Fever sensitivity: 4.6% early vs 20.2% late presenters (> 24 hours of symptoms); aOR for complications 6.64 (95% CI, 1.42*–*31.21; P = .02)*.

How does this improve population health?

*Recognizing the low sensitivity of fever in early acute cholecystitis may reduce diagnostic delays and help identify patients at risk for complications*.

Although fever is not part of the Tokyo criteria for acute cholecystitis severity, systemic inflammation markers, such as leukocytosis > 18,000/μL, are included as criteria for moderate severity (Grade II).^7^ Since fever often accompanies elevated leukocyte counts in inflammatory processes, it may be logical to consider fever as a supplementary indicator of severity. A few studies have described the association between fever and surgical complications or difficult laparoscopic cholecystectomy.^10^–^12^

### Goals of This investigation

The aim of this study was to determine the prevalence of fever as a diagnostic parameter for acute cholecystitis in a population with a high incidence of the condition, according to the symptom onset at the time of medical consultation (≤ 24 hours versus > 24 hours), and its potential association with both medical and surgical complications.

## METHODS

### Study Design and Setting

This was a retrospective cohort study conducted in two emergency departments (ED) of two Chilean urban, tertiary academic hospitals. The study spanned from March 2023 to June 2024. This study was approved by the ethics review board of the participating center and conducted in accordance with local legal and institutional requirements, as well as generally accepted ethical principles including the Declaration of Helsinki, the Belmont Report, and the Nuremberg Code.

### Selection of Participants

Participants included all patients older than 18 years who were consecutively admitted to the hospital from the ED with a clinical diagnosis of acute cholecystitis determined by the treating physician and based on the patient’s clinical presentation and imaging findings in accordance with the Tokyo criteria. Presentations were categorized as “early” if symptom onset occurred within 24 hours prior to arrival to the ED and “late” if symptom onset occurred longer than 24 hours before arrival. The cut point of 24 hours was based on a prior unpublished quality improvement project that described the timing of consultation in the ED for patients diagnosed with acute cholecystitis. In that study, more than half of patients visited the ED before 24 hours of symptoms onset, representing a relevant group of patients that need assessment and risk stratification in the ED. This cut-point also fits with our hypothesis that as early as 24 hours after symptoms onset, obstruction is what leads the clinical scenario and not inflammation.

Screening for inclusion was based on ED discharge diagnoses of acute cholecystitis, biliary colic, cholelithiasis, acute abdomen, sepsis, or septic shock. Including acute abdomen and sepsis/shock ensured inclusion of severe cases with biliary etiology that might otherwise be overlooked on initial evaluation. Exclusion criteria included patients with a final diagnosis other than acute cholecystitis at hospital discharge, those without abdominal pain on presentation and those without a documented time of symptom onset. The cohort aimed to reflect a wide range of disease severity among patients with acute cholecystitis. For the primary outcome the gold standard for the final diagnosis of acute cholecystitis was established by histopathological analysis of the resected gallbladder specimen.

### Interventions

In Chile, cholelithiasis and gallbladder cancer are prevalent, making cholecystectomy the standard of care for patients with symptomatic cholelithiasis, regardless of presentation.^13^,^14^ Medical management is rarely employed, except in cases where proportional care is deemed appropriate due to patient comorbidities or clinical limitations. Thus, all patients followed diagnosis and management according to hospital and national protocols, considering patient stability and clinical judgment of the surgical team on shift.

### Measurements

We collected data from the electronic health record (EHR) including patient demographics, clinical history, physical examination findings, laboratory values, and imaging results. Fever was defined as either a reported history of fever at home or an axillary temperature > 37.5 °C recorded in the ED. This temperature cutoff was selected based on the lowest temperature reported in the literature for evaluating inflammatory criteria in the diagnosis of acute cholecystitis.^15^,^16^ While this cutoff does not align with the threshold established by the Infectious Diseases Society of America, which is a temperature higher than 38.3 °C, it increases sensitivity and allows for comparison with data published in the field.^17^ Additionally, we report alternative cutoffs to broaden the scope of our analysis. Temperature measurements were not standardized due to the retrospective design of the study; axillary temperature was used as it was the most consistently documented method in both prehospital and ED settings. Data on antipyretic use were not systematically recorded in the medical records and were therefore not included in the analysis.

Laboratory data, including C-reactive protein (CRP) and white blood cell (WBC) counts, were categorized as normal based on local laboratory cutoffs (CRP < 0.5 mg/dL, WBC < 11,000/μL). These parameters were measured upon presentation and documented in the EHR. Data abstraction procedures followed methodological recommendations for retrospective medical record review studies as outlined by Worster and Bledsoe.^18^ These included the use of predefined inclusion and exclusion criteria, standardized data collection with operational definitions specified a priori, and trained reviewers who abstracted data from the EHR.

### Outcomes

The primary outcome was the sensitivity of fever for the diagnosis of acute cholecystitis, stratified by early versus late presentation. Sensitivity was defined as the proportion of patients with histopathologically confirmed acute cholecystitis who presented with fever. Secondary outcomes included the sensitivity of other inflammatory markers in early and late presenters and the association between fever and surgical or medical complications. Medical complications were defined as sepsis, septic shock, need for vasopressors, or death, while surgical complications included empyema, gangrene, perforation, need for open conversion, need for drainages, and partial cholecystectomy. For the purposes of this study, clinically diagnosed acute cholecystitis was defined as a final hospital discharge diagnosis established by the treating team based on clinical presentation and compatible imaging findings (ultrasound, CT, or MRI), in accordance with the Tokyo criteria.

### Analysis

Sample-size calculations were based on an estimated sensitivity of 30% in late-presenting patients, reflecting findings from recent literature on fever rates in acute cholecystitis.^8^ For early presenting patients, fever was expected to have a sensitivity of 10%, based on a prior unpublished quality improvement project of patients diagnosed with acute cholecystitis at the ED. This required a sample of 89 patients per group (total 178) to detect a 20% difference with 90% power. Statistical analyses for the primary and secondary outcomes included chi-square tests for categorical variables and Student *t* test or Mann-Whitney-Wilcoxon tests for continuous variables based on distribution. A parsimonious multivariable model was prespecified based on clinical reasoning, including fever as the primary exposure and age and C-reactive protein (CRP) as covariates. Given the limited number of outcome events, variables were restricted to minimize overfitting, and a penalized likelihood logistic regression model (Firth method) was used to reduce small-sample bias. Age and CRP were analyzed as scaled continuous variables (per decade increase and per 10 mg/dL increase, respectively) to improve interpretability.

A complete-case analysis was conducted, and observations with missing data were excluded from the regression models. Analyses were performed using Stata 17 (StataCorp).

## RESULTS

### Main Results

#### Characteristics of Study Subjects

A total of 94,763 patient encounters were screened for diagnoses of acute cholecystitis, biliary colic, cholelithiasis, acute abdomen, and sepsis at the time of admission at the ED or transfer to the ward, yielding 419 eligible records. We excluded 212 encounters (Figure).

Of the included cohort of 207 patients with clinically diagnosed acute cholecystitis, 108 (52.2%) presented within 24 hours of symptom onset (early presentation), and 99 (47.8%) after 24 hours (late presentation). The cohort was 55% female overall, with 58% female in the early presentation group and 52% in the late presentation group (*P* = .35). The median age of the total cohort was 53 years (interquartile range [IQR] 39–64), with a median of 50 years (IQR 38–63) in the early presentation group and 56 years (IQR 42–68) in the late presentation group (*P* = .03). Of those presenting late, 32% presented between 24 and 48 hours of symptoms onset; 21% between 48 and 72 hours, 18% from 72 to 96 hours, and 29% after 96 hours. The median duration of symptoms in the early group was 8.5 hours (IQR 5–18) compared to 72 hours (IQR 48–144) in the late group (*P* < .001). Acute cholecystitis was clinically confirmed with ultrasound in 82% of cases, with CT in 14.5% of cases, and with MRI/MRCP in 2.9% of cases. A total of 141 patients (68%) had histologically confirmed acute cholecystitis, while the remaining 32% of cases had signs of chronic cholecystitis. We found that 55% of early presenters and 81% of late presenters had histologically confirmed acute cholecystitis. Table 1 provides an overview of the clinical characteristics of the analyzed cohort.

### Fever and Clinical Characteristics

The mean axillary temperature on ED arrival was 36.4 °C (0.4). Across all fever cutoffs, including history of fever at home, fever was more prevalent among late-presenting patients. The prevalence of history of fever and/or temperature higher than 37.5 °C in the ED in the early presentation group was 4.6% and 20.2% among late presenters. The clinical description of patients with fever in comparison with patients without is described in Table 2.

### Sensitivity of Tokyo Criteria

The sensitivity of the Tokyo criteria, including fever by early and late presenters is shown in Table 3. Temperature higher than 37.5 °C at ED arrival plus history of fever at home had a sensitivity of 12.1% (95% CI, 8.0–17.2), with lower sensitivity in early presenters (4.6% [95% CI, 1.5–10.4]) compared to late presenters (20.2% [95% CI, 12.8–29.4]). Fever, defined as higher than 38 °C in axillary temperature in the ED, had a sensitivity of 1.5% [95% CI, 0.3–4.3] overall in this cohort, with a 0% (exact 95% CI, 0.0–3.4) for early presenter and 3% (95% CI, 0.6–8.5) in late presenters.

### Complications

Fifteen patients experienced complications. Two patients presented with medical complications, one of whom had fever in the ED. Patients with fever, defined as history of fever at home and/or axillary temperature higher than 37.5 °C (*n* = 25), had higher rates of surgical complications (24.0% in febrile versus 3.9% in afebrile patients; odds ratio [OR] 4.81; 95% CI, 1.03–22.37; *P* < .001). Among the group of patients with a temperature higher than 38 °C, one required vasopressors for sepsis, another had concurrent choledocholithiasis requiring a laparoscopic rendezvous procedure, and a third developed a hepatic abscess requiring drainage.

In the complete cohort, a history of fever and/or an axillary temperature higher than 37.5 °C (12.1%, *n* = 25) was significantly associated with complications in a penalized logistic regression model. The adjusted OR for complications associated with fever was 6.64 (95% CI, 1.42–31.21; *P* = .02) adjusted for CRP (modeled per 10 mg/dL increase) and age (modeled per decade increase) (Table 4). C-reactive protein was independently associated with complications (aOR 2.26 per 10 mg/dL increase; 95% CI, 1.24–4.11; *P* = .01), as was age (aOR 3.03 per 10-year increase; 95% CI, 1.63–5.63; *P* < .001). In subgroup analyses, the aOR for complications associated with fever was 20.5 (95% CI, 0.54–777.34; *P* = .10) among early presenters and 4.38 (95% CI, 0.80–23.86, *P* = .09) among late presenters.

## DISCUSSION

### Interpretation

Our findings align with studies by Naidu et al and Jain et al, which demonstrate limited sensitivity of fever as a diagnostic criterion for acute cholecystitis.^8^,^9^ Notably, the sensitivity of fever varies significantly depending on the timing of symptom onset—only reaching 1.9% (95% CI, 0.2–6.8) in early presentations and 7.1% (95% CI, 2.9–14.1) in late presentations when considering 37.5 °C as the cutpoint. These findings suggest that systemic inflammatory signs may not be reliably present in the initial phase of disease.

Leukocytosis and CRP were more frequently abnormal than fever; however, they also demonstrated only moderate sensitivity in early presentations. Together, these observations highlight the potential limitations of relying on systemic inflammatory markers as diagnostic signals in the hyperacute phase of acute cholecystitis. From a prognostic perspective, fever was associated with complications after adjustment for age and CRP (OR 6.64; 95% CI, 1.42–31.21). C-reactive protein (modeled per 10 mg/dL increase) and age (per decade increase) were also independently associated with complications. However, the confidence intervals were wide, reflecting the limited number of outcome events, and preclude definitive conclusions regarding the magnitude of effect.

### Summary of Main Findings

In this retrospective cohort of patients with clinically diagnosed acute cholecystitis, fever was uncommon at presentation—even at lower rates than those reported in the literature—and demonstrated very low sensitivity as a diagnostic criterion (histological confirmation), particularly among patients presenting within 24 hours of symptom onset.^8^,^19^ Even when combining measured temperature and history of fever at home, overall sensitivity remained limited. Although fever was infrequent, it was associated with complications after adjustment for age and CRP using a penalized likelihood logistic regression model. Given the small number of outcome events, these findings should be interpreted cautiously and considered exploratory.

### Comparison to Previous Studies

Although this study was not powered to evaluate the predictive value of fever as a marker of severity in acute cholecystitis, the presence of fever may serve as a specific marker for severe disease and could complement other systemic inflammation indicators. An axillary temperature higher than 37.5 °C or a patient-reported history of fever was independently associated with complications after adjusting for CRP and age in a penalized logistic regression model. Since the number of complications was small in our study, it may be necessary to explore these results in a larger study. Nonetheless, to our knowledge, the 207 patients included here represent one of the largest samples specifically examining fever characteristics in relation to clinical outcomes in acute cholecystitis, which strengthens the precision and reliability of the descriptive estimates across different stages of symptom onset.

### Strengths

Strengths of this study include histopathologic confirmation of acute cholecystitis, detailed characterization of symptom timing, and the use of penalized logistic regression to mitigate small-sample bias. The stratification by timing of presentation provides clinically interpretable insights into early versus later manifestations of systemic inflammation.

### Clinical implications

From an ED perspective, these findings suggest that the absence of fever should not be used to exclude acute cholecystitis, particularly in patients presenting early in the course of disease. In this setting, reliance on fever as a diagnostic criterion may delay diagnosis and imaging. Instead, clinical suspicion should be guided primarily by local signs and symptoms, with inflammatory markers providing complementary but limited diagnostic support.

In patients presenting with fever and clinically diagnosed acute cholecystitis, alternative diagnoses such as cholangitis, pyelonephritis, or other infectious sources should be reconsidered. Alternatively, the presence of fever may indicate that the patient is at elevated risk for medical and surgical complications from acute cholecystitis, alerting the emergency physician, which may evaluate appropriate management strategies, including prompt surgical consultation and possible disposition to a higher level of care.

### Research Implications

Future research should prospectively evaluate fever as a predictor of complications in larger, multicenter cohorts. Studies incorporating continuous temperature monitoring and longitudinal inflammatory markers may better characterize the temporal relationship between fever onset and disease progression in acute cholecystitis.

## LIMITATIONS

This was a retrospective two-center study, which may limit generalizability. The high prevalence of cholelithiasis in the Chilean population may influence disease presentation and diagnostic practices. The number of complications was small, limiting statistical power and resulting in wide confidence intervals, particularly in subgroup analyses. Although we adjusted for age and CRP, residual confounding remains possible. We were unable to account for additional physiologic parameters such as blood pressure, shock index, markers of organ dysfunction, or other laboratory indicators that may influence risk stratification. Additionally, history of fever at home was self-reported and may be subject to recall bias. However, this information remains relevant to real-world clinical decision-making and risk stratification.

Prior studies have shown that self-reported fever may have moderate diagnostic value in identifying infectious conditions, like pneumoniae and influenza and is frequently used as part of initial clinical assessment in the emergency department.^20^,^21^ Kass Hout et al found a moderate agreement between measured and self-reported fever (*k*, 0.423; 95% CI, 0.420–0.425).^22^ In this context, patient-reported fever may serve as a complementary clinical signal that reflects early systemic inflammatory response, but it should be interpreted cautiously and in conjunction with other clinical and laboratory findings.

We stratified patients using a 24-hour threshold from symptom onset to distinguish early from later clinical presentations. This cutoff was selected to reflect a clinically meaningful window in emergency care decision-making and to approximate the early inflammatory phase of disease evolution. However, inflammatory progression is inherently continuous, and dichotomization may oversimplify the biological process.

## CONCLUSION

In this retrospective cohort, fever demonstrated low sensitivity as a diagnostic sign of acute cholecystitis, particularly in early presentations. Although an association with complications was observed after adjustment for age and C-reactive protein, the limited number of outcome events and study design preclude firm conclusions regarding its prognostic value. These findings suggest that fever has limited diagnostic utility in early acute cholecystitis and may warrant further investigation as a potential marker of severity in larger prospective studies.

## Figures and Tables

**Figure:**
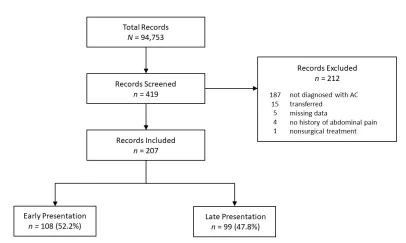
Study flowchart for a retrospective cohort of 207 patients with acute cholecystitis in a study to determine the prevalence of fever as a diagnostic parameter for the disease. Early presentation: within 24 hours of symptom onset. Late presentation after 24 hours. *AC*, acute cholecystitis.

**Table 1:** Clinical characteristics, laboratory findings, and imaging results of patients with clinically diagnosed acute cholecystitis, stratified by time from symptom onset.

	Total (*N* = 207)	Early Presentation (*n* = 108, 52.2%)	Late Presentation (*n* = 99, 47.8%)	Group comparison
Clinical variables				
RUQ pain, *n* (%)	162 (78.3%)	77 (71.3%)	85 (85.9%)	*P* = .01
Murphy sign, *n* (%)	74 (35.8%)	30 (27.8%)	44 (44.4%)	*P* = .02
Hx of fever at home, *n* (%)	17 (8.2%)	3 (2.8 %)	14 (14.1 %)	*P* < .001
Temperature (°C), mean (SD)	36.4 (0.5)	36.3 (0.4)	36.6 (0.5)	*P* < .001
Temperature > 37.5 °C, *n* (%)	9 (4.3%)	2 (1.9%)	7 (7.1%)	*P* = .07
Temperature ≥ 37.8 °C, *n* (%)	6 (2.9%)	1 (0.9%)	5 (5.1%)	*P* = .08
Temperature ≥ 38 °C, *n* (%)	3 (1.4%)	0 (0%)	3 (3.0%)	*P* = .07
Hx + Temperature ≥ 37.5 °C, *n* (%)	25 (12.1%)	5 (4.6%)	20 (20.2%)	*P* < .001
Hx + ≥ Temperature ≥ 37.8 °C, *n* (%)	22 (10.6%)	4 (3.7%)	18 (18.2%)	*P* < .001
Hx + Temperature ≥ 38 °C, *n* (%)	19 (9.2%)	3 (2.8%)	16 (16.2%)	*P* < .001
Laboratory				
CRP (mg/dL), median	1.3 [0.3–10.6]	0.4 [0.2–1.0]	10.1 [2.5–18.4]	*P* < .001
CRP ≥ 0.5 (mg/dL)	144 (69.6%)	52 (48.1%)	92 (92.9%)	*P* < .001
WBC (x10^3^/μL), mean (SD)	11.6 (4.1)	11.5 (4.4)	11.6 (3.8)	*P* = .90
WBC ≥ 11.0 (x10^3^/μL), *n* (%)	114 (55.1%)	57 (52.8%)	57 (57.6%)	*P* = .49
Imaging				
Abdominal ultrasonography, *n* (%)	166 (80.2%)	95 (88.0%)	71 (71.7%)	
Abdomen-pelvis contrasted CT, *n* (%)	30 (14.5%)	10 (9.3%)	20 (20.2%)	
MRI, *n* (%)	6 (2.9%)	2 (1.9%)	4 (4.0%)	

Temperature was defined as the axillary temperature at ED arrival.

*CRP*, C-reactive protein; *CT*, computed tomography; *Hx*, history of; *MRI*, magnetic resonance imaging; *RUQ*, right upper quadrant; *SD*, standard deviation; *WBC*, white blood cell count.

**Table 2:** Clinical characteristics of patients with clinically diagnosed acute cholecystitis stratified by the presence of fever.

	Fever (*n* = 25, 12.1%)	No fever (*n* = 182, 87.9%)	Group comparison
Clinical variables			
Age (years), mean (SD)	56 (15)	51.8 (16)	*P* = .21
Female, *n* (%)	11 (44%)	102 (56%)	*P* = .25
Time of symptom onset (hours), median [IQR]	48 [48–72]	24 [6–72]	*P* < .001
Temperature (°C), mean (SD)	37 (0.14)	36 (0.02)	*P* < .001
Laboratory			
CRP (mg/dL), mean (SD)	19.4 (12.9)	5.4 (8.2)	*P* < .001
WBC (x10^3^/μL), mean (SD)	13 (4.6)	11 (4)	*P* = .03
Diagnosis and complications			
Medical complications, *n* (%)	1 (4%)	1 (0.5%)	*P* = .10
Surgical complications, *n* (%)	6 (24%)	7 (3.8%)	*P* < .001
Death, *n* (%)	1 (4%)	0 (0%)	*P* < .001

Fever defined as history of fever at home or axillary temperature at ED arrival > 37.5°C.

*CRP*, C-reactive protein; *ED*, emergency department; *SD*, standard deviation; *WBC*, white blood cell count.

**Table 3:** Sensitivity of Tokyo diagnostic criteria for acute cholecystitis by time of symptom onset in a retrospective cohort study of patients with clinically diagnosed acute cholecystitis.

	Early presenters	Late Presenters	Total
RUQ pain	71.3% [62.0–79.3]	85.9% [77.7–91.8]	78.3% [72.4–83.3]
Murphy sign	27.8% [19.7–37.2]	44.4% [34.6–54.6]	35.7% [29.3–42.6]
T > 37.5°C	1.9% [0.2–6.8]	7.1% [2.9–14.1]	4.4% [2.0–8.1]
Hx + T > 37.5°C	4.6% [1.5–10.4]	20.2% [12.8–29.4]	12.1% [8.0–17.2]
T ≥ 38°C	0.0% [0–3.4]	3.0% [0.6–8.5]	1.5% [0.3–4.3]
Hx + T ≥ 38°C	2.8% [0.6–8.0]	16.2% [9.6–25.0]	9.2% [5.6–14.0]
CRP ≥ 0.5 (mg/dL)	48.2% [38.5–58.1]	92.9% [85.9–97.1]	69.6% [63.0–75.6]
WBC ≥11.0 (x103/μL)	52.8% [43.0–62.5]	57.6% [47.3–67.4]	55.1% [48.1–62.0]

Temperature was defined as the axillary temperature at ED arrival.

*CRP*, C-reactive protein; *Hx*, history of; *RUQ*, right upper quadrant; *WBC*, white blood cell count.

**Table 4:** Penalized logistic regression model of risk factors for complications in patients with acute cholecystitis, adjusted for age and C-reactive protein, in a retrospective cohort study of cases of acute cholecystitis.

Variable	Odds ratio	95% CI	*P* value
Fever	6.64	1.42 – 31.21	.02
CRP (per 10 mg/dL increase)	2.26	1.24 – 4.11	.01
Age (per 10-year increase)	3.03	1.63 – 5.63	< .001

Fever defined as history of fever at home or axillary temperature at ED arrival > 37.5 °C.

*CRP*, C-reactive protein; *ED*, emergency department.

## References

[1] Roslyn JJ, Thompson JE (1980). Roles of lithogenic bile and cystic duct occlusion in the pathogenesis of acute cholecystitis. Am J Surg.

[2] Gallaher JR, Charles A (2022). Acute cholecystitis: a review. JAMA.

[3] Ministerio de Salud de Chile (2014). Guías Clínicas AUGE: Colecistectomía Preventiva en Adultos de 35 a 49 Años.

[4] Kimura Y, Takada T, Kawarada Y (2007). Definitions, pathophysiology, and epidemiology of acute cholangitis and cholecystitis: Tokyo Guidelines. J Hepatobiliary Pancreat Surg.

[5] Moon DK, Kang JS, Byun Y (2023). Incidence of bactibilia and related factors in patients who undergo cholecystectomy. Ann Surg Treat Res.

[6] Morris-Stiff GJ, O’Donohue P, Ogunbiyi S (2007). Microbiological assessment of bile during cholecystectomy: Is all bile infected?. HPB (Oxford).

[7] Yokoe M, Hata J, Takada T (2018). Tokyo Guidelines 2018: Diagnostic criteria and severity grading of acute cholecystitis (with videos). J Hepatobiliary Pancreat Sci.

[8] Naidu K, Beenen E, Gananadha S (2016). The yield of fever, inflammatory markers and ultrasound in the diagnosis of acute cholecystitis: A validation of the 2013 Tokyo Guidelines. World J Surg.

[9] Jain A, Mehta N, Secko M (2017). History, physical examination, laboratory testing, and emergency department ultrasonography for the diagnosis of acute cholecystitis. Acad Emerg Med.

[10] Borzellino G, Steccanella F, Mantovani W (2013). Predictive factors for the diagnosis of severe acute cholecystitis in an emergency setting. Surg Endosc.

[11] Stoica PL, Serban D, Bratu DG (2024). Predictive factors for difficult laparoscopic cholecystectomies in acute cholecystitis. Diagnostics (Basel).

[12] Halachmi S, DiCastro N, Matter I (2000). Laparoscopic cholecystectomy for acute cholecystitis: How do fever and leucocytosis relate to conversion and complications?. Eur J Surg.

[13] Nervi OF (2001). Cáncer de la vesícula biliar en Chile. Rev Med Chil.

[14] Miquel JF, Covarrubias C, Villaroel L (1998). Genetic epidemiology of cholesterol cholelithiasis among Chilean Hispanics, Amerindians, and Maoris. Gastroenterology.

[15] Bednarz GM, Kalff V, Kelly MJ (1986). Hepatobiliary scintigraphy: Increasing the accuracy of the preoperative diagnosis of acute cholecystitis. Med J Aust.

[16] Schofield PF, Hulton NR, Baildam AD (1986). Is it acute cholecystitis?. Ann R Coll Surg Engl.

[17] O’Grady NP, Barie PS, Bartlett JG (2008). Guidelines for evaluation of new fever in critically ill adult patients: 2008 update from the American College of Critical Care Medicine and the Infectious Diseases Society of America. Crit Care Med.

[18] Worster A, Bledsoe RD, Cleve P (2005). Reassessing the methods of medical record review studies in emergency medicine research. Ann Emerg Med.

[19] Gruber PJ, Silverman RA, Gottesfeld S (1996). Presence of fever and leukocytosis in acute cholecystitis. Ann Emerg Med.

[20] Metlay JP, Kapoor WN, Fine MJ (1997). Does this patient have community–acquired pneumonia? Diagnosing pneumonia by history and physical examination. JAMA.

[21] Call SA, Vollenweider MA, Hornung CA (2005). Does this patient have influenza?. JAMA.

[22] Kass-Hout TA, Buckeridge D, Brownstein J (2012). Self-reported fever and measured temperature in emergency department records used for syndromic surveillance. J Am Med Inform Assoc.

